# Pulmonary hypertension in the changing landscape of congenital heart disease: Global differences and a possible driver of end-stage heart failure

**DOI:** 10.1007/s12471-016-0844-4

**Published:** 2016-05-12

**Authors:** A. Van De Bruaene, W. Budts

**Affiliations:** Congenital and Structural Cardiology, University Hospitals Leuven and Department of Cardiovascular Sciences, Catholic University Leuven, Leuven, Belgium

In this issue of the Netherlands Heart Journal, the articles of Van Riel et al. and Couperus et al. focus on some of the questions encountered in contemporary clinical congenital cardiology practice [1, 2].

Pulmonary arterial hypertension associated with congenital heart disease (PAH-CHD) is rare, with an estimated prevalence between 1.6 and 12.5 cases per million adults [3]. Registries have provided important information on the epidemiology and clinical phenotype of patients with PAH-CHD [4–6], but have often been limited to a Western, white patient population, limiting comparison between population demographics and healthcare delivery systems. The article by Van Riel et al. provides valuable new information [2]. They showed that despite having worse exercise performance at baseline, Singaporean patients not only had similar improvement on exercise capacity, but even better survival when compared with their Dutch counterparts. Age at initiation of disease-targeting therapy (either phosphodiesterase-type-5 inhibitors, endothelin receptor antagonists, or a combination) was the strongest predictor of treatment efficacy and outcome [2].

Although the authors subsequently state that early initiation of (combination) disease-targeting therapy is important for outcome, which is probably correct and supported by other studies [7, 8], the observed difference may also reflect a changing phenotype of the patient with PAH-CHD. Increased awareness, better screening tools and an ageing population have shifted demographics in idiopathic PAH registries from a younger, predominantly female patient population in the first US National Institutes of Health (NIH) registry to an older patient population with a more equal gender distribution in more contemporary registries [9–11]. In contrast, registries from China still show demographics similar to the early US NIH studies [12]. Similarly, it is likely that due to improved survival of moderate and complex CHD, increased awareness for PAH, and ageing of the CHD population in Western countries, the profile of the adult PAH-CHD patient has changed. More patients will have PAH-CHD which developed after (and despite of) one or multiple surgical procedures rather than the ‘classical form’ of Eisenmenger’s syndrome due to unrepaired shunt lesions at birth [6, 13].

This change in phenotype of PAH-CHD is nicely highlighted in the article by Couperus et al. which provides a fascinating insight into the tailored treatment of some of the patients presenting with pulmonary hypertension after having had surgical repair or palliation in the past [1]. Although the current guidelines definitively provide a handhold [14], it is undeniable that some cases fall between the cracks of these guidelines. In their article, Couperus et al. describe two patients who had progressive right ventricular failure due to pulmonary hypertension (one in the presence of a recanalised central shunt with left-to-right shunting), one patient with worsening cyanosis in the setting of a hypoplastic pulmonary vasculature resulting in pulmonary hypertension with right-to-left shunting and one with pulmonary hypertension associated with increased left atrial pressure due to atrioventricular valve stenosis in the setting of a univentricular heart [1]. It is clear that all four patients had heart failure with pulmonary (arterial) hypertension being the main driver of the heart failure symptoms, but their specific cases may not be covered in detail by recently published recommendations for CHD heart failure [15]. It is also immediately clear that heart failure in adults with CHD is vastly different from ‘classic’ heart failure in the general population, developing at an earlier age (patients were 23 to 49 years of age at the time of intervention [1]), and often with an underlying ‘heart failure-inducing’ haemodynamic lesion. In such circumstances surgery or percutaneous interventions may improve heart failure, even in the setting of pulmonary hypertension. It is clear that in the future there will be a need for specialised heart failure units for adult CHD patients, with close communication and collaboration between adult CHD specialists, heart failure specialists (indication for assist device; transplantation), cardiac surgeons (indication for assist devices; transplantation; intervention), interventional cardiologists, imaging and pulmonary hypertension specialists to care for those patients with complex PAH-CHD.

Registries are crucial in order to describe the clinical phenotype and outcome of patients with PAH-CHD to extend knowledge and to serve as a base for future research hypotheses. A global approach helps to increase the number of patients studied and to understand the impact of ethnicity in PAH-CHD. A change in the phenotype of the PAH-CHD patient is reflected in a growing number of complex CHD patients with a subsequent increase in the prevalence of pulmonary hypertension. Often, rather than the prototypical Eisenmenger patient, these are heart failure patients in whom pulmonary hypertension has become the driver of their heart failure symptoms. An adult CHD specialist with understanding of the underlying cardiac anatomy, the physiology of pulmonary hypertension, and the triggers of heart failure is mandatory in the teams that participate in the care of these kinds of patients.
